# Supplementary material for: Regulation of molecular transport in polymer membranes with voltage-controlled pore size at the angstrom scale
Source: Nat Commun. 2023 Apr 25;14:2373. doi: 10.1038/s41467-023-38114-3 (PMC10130050; doi:10.1038/s41467-023-38114-3)
Supplement: Supplementary file 1 — Supplementary Information [file 41467_2023_38114_MOESM1_ESM.pdf]

Supplementary Information

Regulation of Molecular Transport in Polymer Membranes with Voltage-controlled Pore Size at the Angstrom Scale

Yuzhang Zhu, et al.

Table of Contents

1 Supplementary Methods 1
2 Supplementary Figures 2
2.1 Surface morphology and chemical composition of the CNTs-PA membrane...................... 2
2.2 Electrically regulating the PEG rejection of the CNTs-PA membranes............................... 5
2.3 Electrically regulating the pore size of silver-PA membrane 7
2.4 Electrically regulating the dyes rejection and desalination performance of the CNTs-PA membrane.. 9
2.5 Cyclic voltammetry measurement and actual membrane potential 11
2.6 Pore size regulation of the CNT-PA membrane working as an anode 13
2.7 Pore size regulation of MPIP based CNTs-PA membrane by the applied voltage............. 14
2.8 Pore size regulation of the CNTs-PA membrane by the voltage in pure water 17
2.9 Partition coefficient of counter ions based on the Donnan equilibrium 17
3 Supplementary Tables.. 18
4. Supplementary References.. 20

1 Supplementary Methods

The materials used in this work. 1,3,5-Trimesoyl chloride (TMC, 98%), 2-methylpiperazine (MPIP, 99%) and anhydrous piperazine (PIP, 99%) were purchased from Aladdin Co., Ltd. (Shanghai, China). Polyether sulfone (PES) microfiltration (MF) membrane (0.22 μm) were commercially available from Yibo Co., Ltd. (Haining, Zhejiang province, China). Polyethylene glycol (PEG, MW = 200, 400, 600 and 1000 Da) was provided by Sinopharm Chemical Reagent Co., Ltd (Shanghai, China). Hydroxyl-functionalized multi-walled carbon nanotubes (CNTs, the content of OH is 5.38 wt%) and silver nanowires were obtained from Nanjing XFNANO Materials Tech Co., Ltd. (Nanjing, China). Na_2SO_4 (≥ 99%), K_2SO_4 (≥ 99.5%), $MgCl_2$ (≥ 99.99%), $CaCl_2$ (≥ 97%), LiCl (≥ 99%), NaCl (≥ 99%) and anhydrous n-hexane were purchased from Sinopharm Chemical Reagent Co., Ltd (Shanghai, China). All chemical reagents were used as received without further purification. Deionized water was used throughout the whole experiment.

2 Supplementary Figures

2.1 Surface morphology and chemical composition of the CNTs-PA membrane

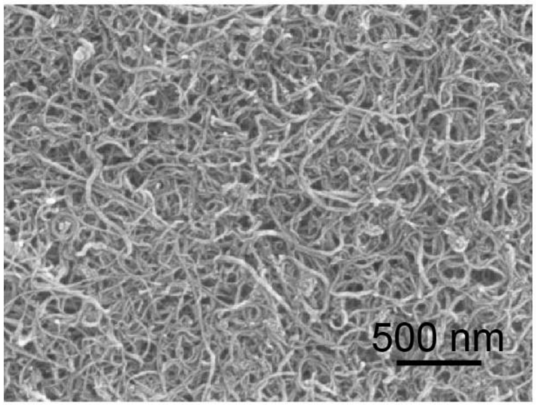

Supplementary Fig. 1. Surface SEM image of CNTs support nanofilm. The loading mass of the hydroxyl-functionalized MWCNT is 0.084 mg cm^{-2}.

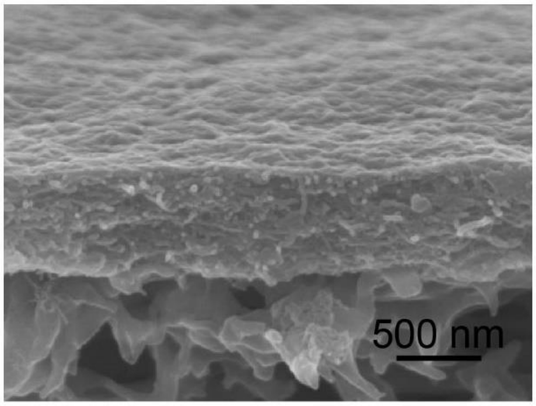

Supplementary Fig. 2. Cross-sectional SEM image of the CNTs-PA membrane. As measured from the SEM image, the thickness of the CNTs layer is around 560 nm, which is consistent with the results observed from the cross-sectional TEM image shown in Fig. 2c in the main text.

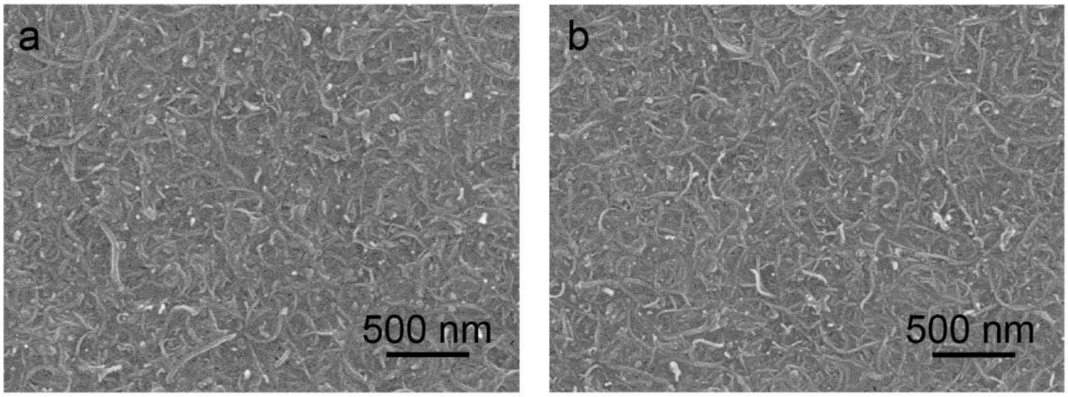

Supplementary Fig. 3. Surface SEM images of the CNTs-PA membrane (a) before and (b) after being used as a cathode at 1.0 V.

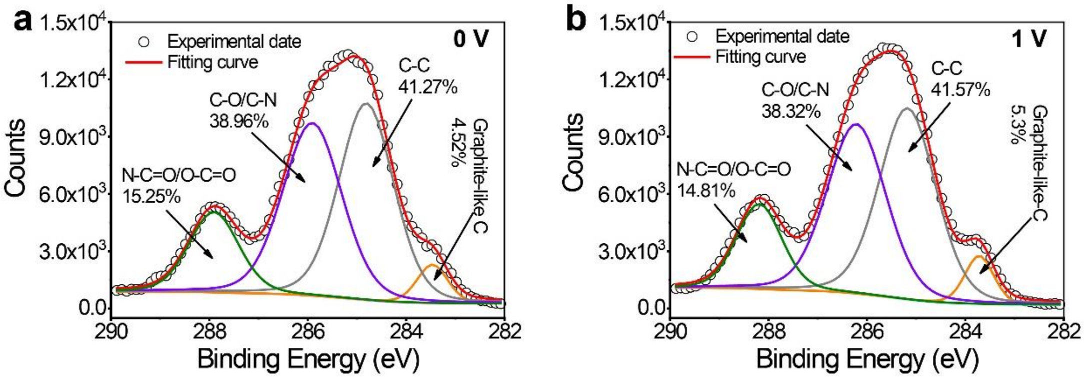

Supplementary Fig. 4. Surface chemical composition of CNTs-PA membrane characterized by the XPS C 1s spectra (a) before and (b) after being used as a cathode at 1.0 V.

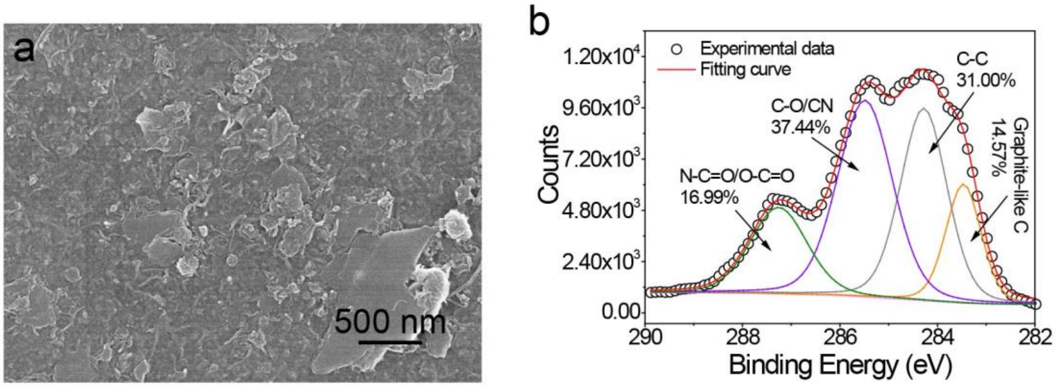

Supplementary Fig. 5. (a) Surface SEM image and (b) XPS C1s spectra of CNTs-PA membrane after being used as a cathode at a voltage of 1.5 V.

Note: From the SEM images displayed in Supplementary Fig. 3, we found little difference in the membrane surface morphology before and after being used as a cathode at 1.0 V. Correspondingly, the content of the surface chemical composition of the membrane determined by the XPS C1s spectra had little difference in this case (Supplementary Fig. 4). In contrast, obvious destruction of the surface morphology occurred when a voltage of 1.5 V was applied to the CNTs-PA membrane (Supplementary Fig. 5). Meanwhile, the chemical composition of CNTs-PA membrane presented apparent change as well. Therefore, we chose 0-1.0 V as the safe range of voltage to study the effect of voltage on the pore size of the CNTs-PA membrane.

2.2 Electrically regulating the PEG rejection of the CNTs-PA membranes

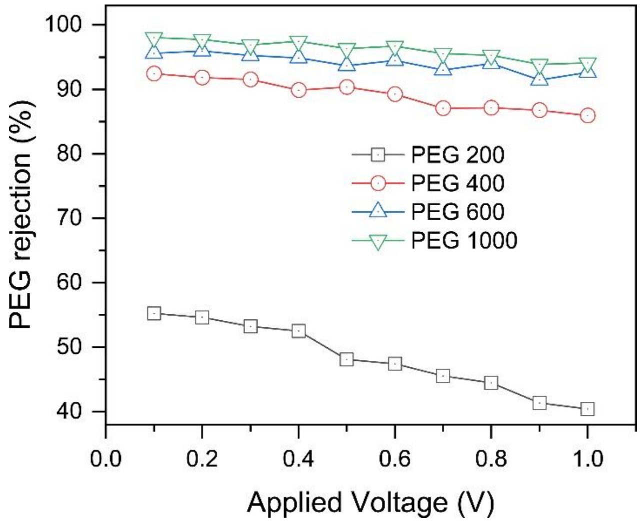

Supplementary Fig. 6. The rejections of PEG with different molecular weights by the CNTs-PA membrane as a function of applied voltage in the presence of 1000 ppm Na_2SO_4.

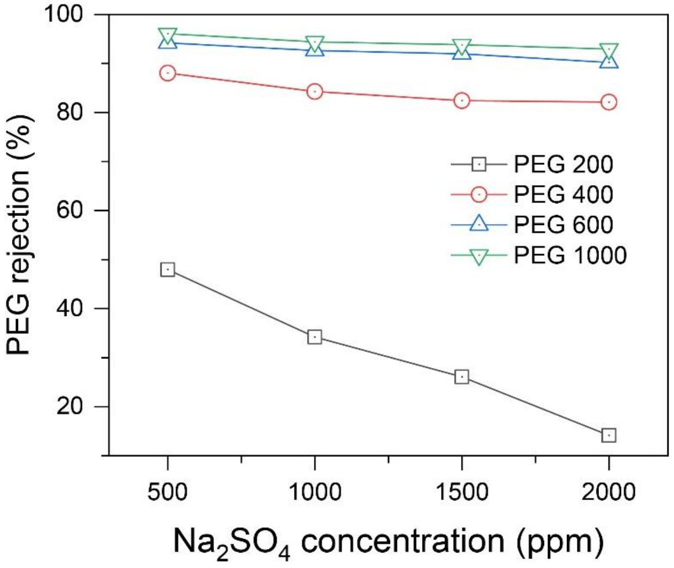

Supplementary Fig. 7. The rejections of PEG with different molecular weights by the CNTs-PA membrane as a function of Na_2SO_4 concentration. The applied voltage was 1.0 V using the membrane as a cathode.

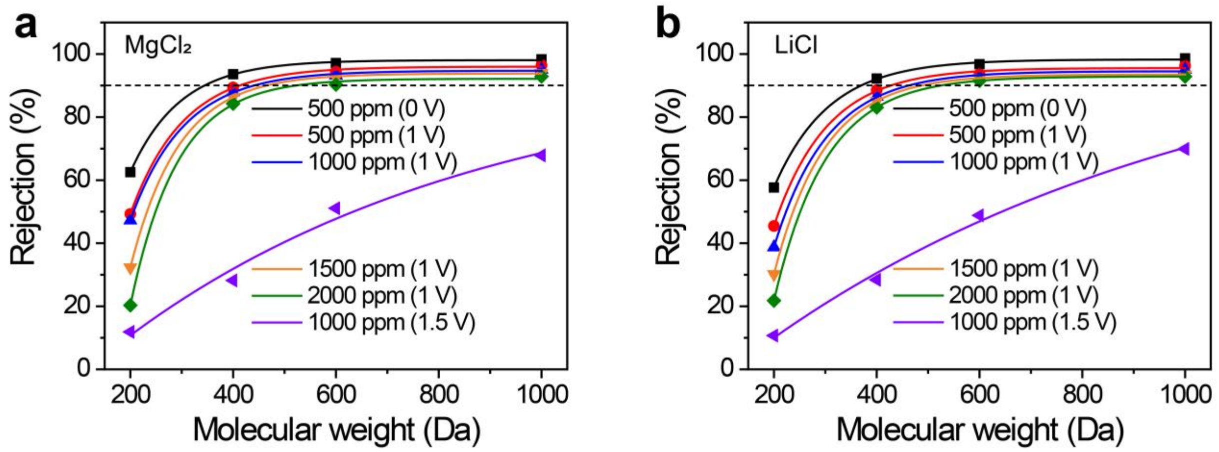

Supplementary Fig. 8. The PEG rejections by the CNTs-PA membrane when varying the voltages and the concentration of electrolyte solution. (a) The electrolyte is $MgCl_2$. (b) The electrolyte is LiCl.

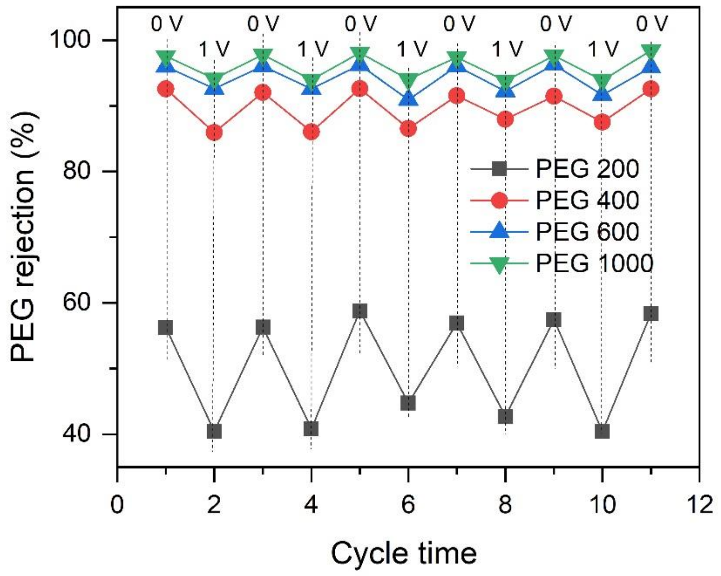

Supplementary Fig. 9. The PEG rejections with different molecular weight by the CNTs-PA membrane during the cycling experiment by switching the applied voltage between 0 V and 1.0 V 11 times in the presence of 1000 ppm Na_2SO_4.

2.3 Electrically regulating the pore size of silver-PA membrane

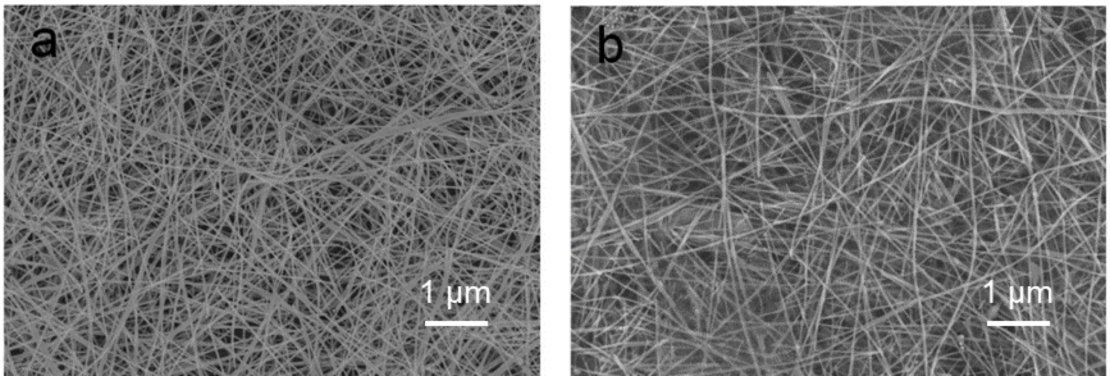

Supplementary Fig. 10. Surface SEM images of (a) silver nanowires nanofilm support and (b) silver-PA membrane.

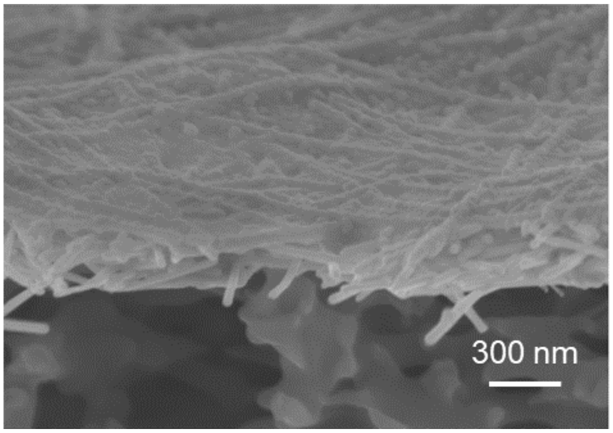

Supplementary Fig. 11. Cross-sectional SEM image of silver-PA layer.

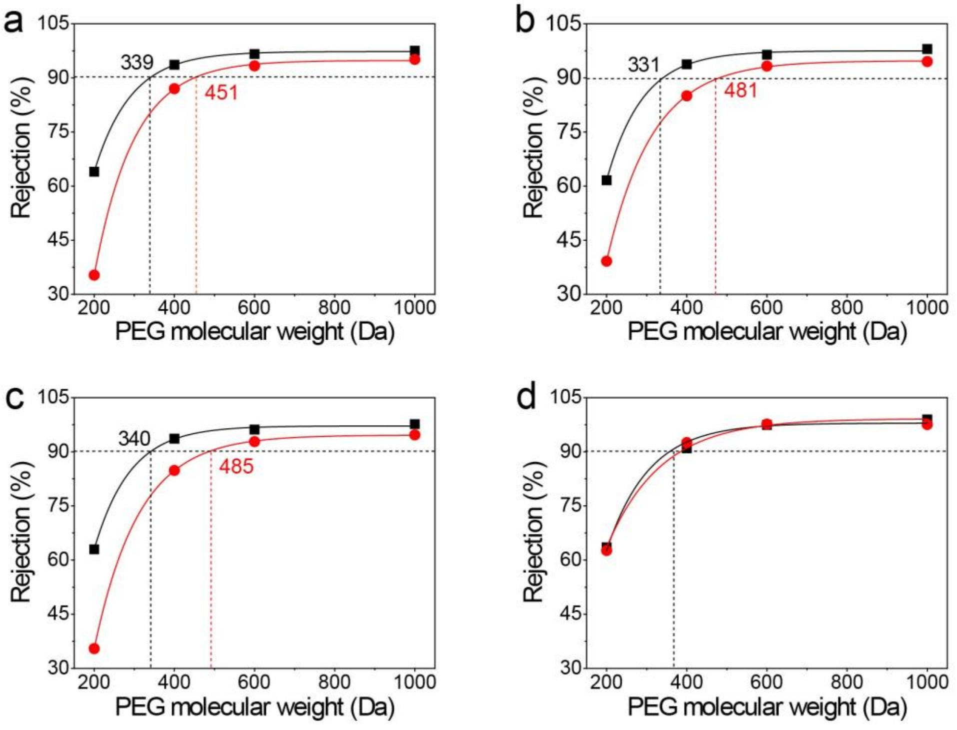

Supplementary Fig. 12. The PEG rejection with different molecular weight by the silver-PA membrane being used as the cathode at 0 V (black line) and 1.0 V (red line). The measurement was performed in the presence of (a) 1000 ppm Na_2SO_4, (b) 1000 ppm $MgCl_2$, (c) 1000 ppm LiCl, and (d) pure water.

Note: Besides hydroxyl-functionalized MWCNTs, conductive silver nanowire was also used to fabricate electrically conductive PA NF membrane. By depositing the silver nanowires onto the surface of PES MF support, silver nanowires nanofilm with interconnected network structure was formed (Supplementary Fig. 10a). Using this silver nanowire nanofilm as support for IP reaction of PIP and TMC, an ultrathin transparent film was formed on its surface after the IP reaction (Supplementary Fig. 10b). Being similar as the geometric structure of the CNTs-PA membrane, the PA layer was embedded in the silver nanowires network (Supplementary Fig. 11), which endow the silver-PA layer with conductive properties. As determined by the PEG rejection (Supplementary Fig. 12), the MWCO of the silver-PA membrane exhibited an obvious increase when used as a cathode in the presence of electrolyte solution (Supplementary Fig. 12a-c). In pure water, the applied voltage has little effect on the MWCO of the silver-PA membrane (Supplementary Fig. 12d), which is also similar to the CNTs-PA membrane. These results strongly support the mechanism of operando pore size tunning by applied voltage is mainly physical.

2.4 Electrically regulating the dyes rejection and desalination performance of the CNTs-PA membrane

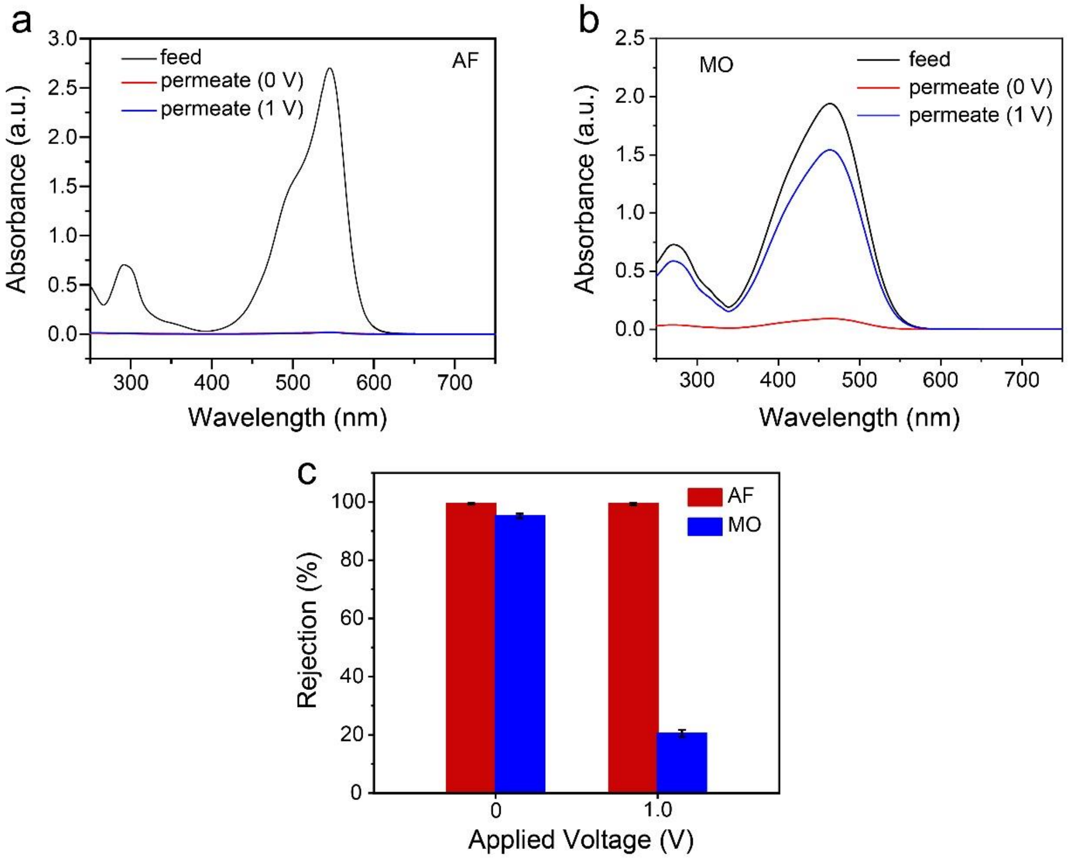

Supplementary Fig. 13. Rejections of acid fuchsin (AF, MW = 585.5 Da) and methyl orange (MO, MW = 327.3 Da) by the CNTs-PA membrane at 0 V and 1.0 V. (a) UV-vis absorption spectra of the AF feed solution and the corresponding permeate collected at 0 V and 1.0 V. (b) UV-vis absorption spectra of the MO feed solution and the corresponding permeate collected at 0 V and 1.0 V. (c) Rejections of AF and MO calculated from the corresponding UV-vis absorption spectra.

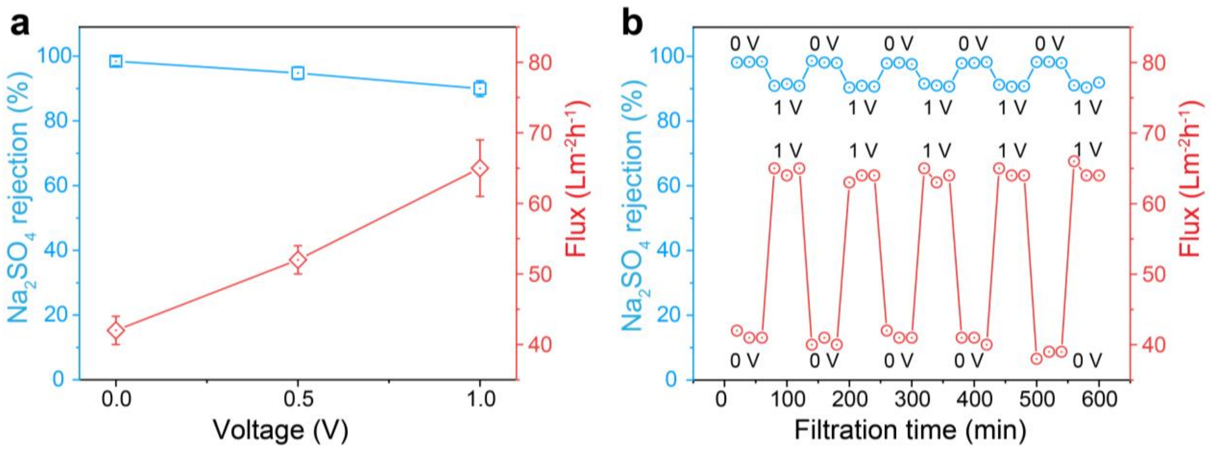

Supplementary Fig. 14. (a) The variation of Na_2SO_4 rejection and permeating flux with the increase of the applied voltage when using CNTs-PA membrane as a cathode. (b) The continuous variation of Na_2SO_4 rejection and permeating flux by switching the voltage from 0 V to 1 V in the cycling experiment.

Note: The rejection of the CNTs-PA membrane was tested according to the methodology reported in our early work[1-3]. A crossflow apparatus with a circular cell was used to test the membrane performance at room temperature. The effective filtration area of the cell is 7.1 cm^2. The applied pressure is 4 bar for desalination. The salt rejection (R) was calculated from the conductivity of feed and permeate according to Eq. S1, where C_f and C_p represent the conductivity of feed and permeate solutions, respectively.

$$R = \left(1 - \frac{C_p}{C_f}\right) \times 100\% \qquad (S1)$$

The flux J is determined by Eq. S2, where Δw is the weight of collected permeate within the filtration time Δt, A is the effective separation area of the cell, ρ is the density of permeate (being considered as 1 g/ml).

$$J = \frac{\Delta w}{\rho A \Delta t} \qquad (S2)$$

2.5 Cyclic voltammetry measurement and actual membrane potential

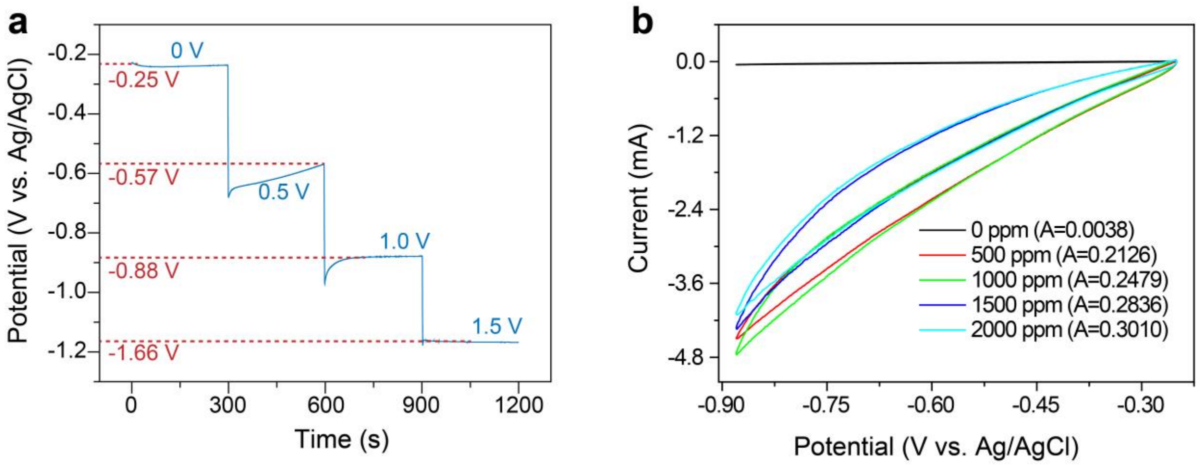

Supplementary Fig. 15. (a) Surface potentials of the CNTs-PA membrane vs. the reference electrode (Ag/AgCl) being used it as cathode at different applied voltages in the presence of 1000 ppm Na_2SO_4 aqueous solution. (b) CV curves of the CNTs-PA membrane being used as the cathode in the presence of Na_2SO_4 aqueous solution with different concentrations. A represents the integral area of the CV curve. The scanning potential range was determined by the open circuit potential measurement shown in Supplementary Fig. 15a.

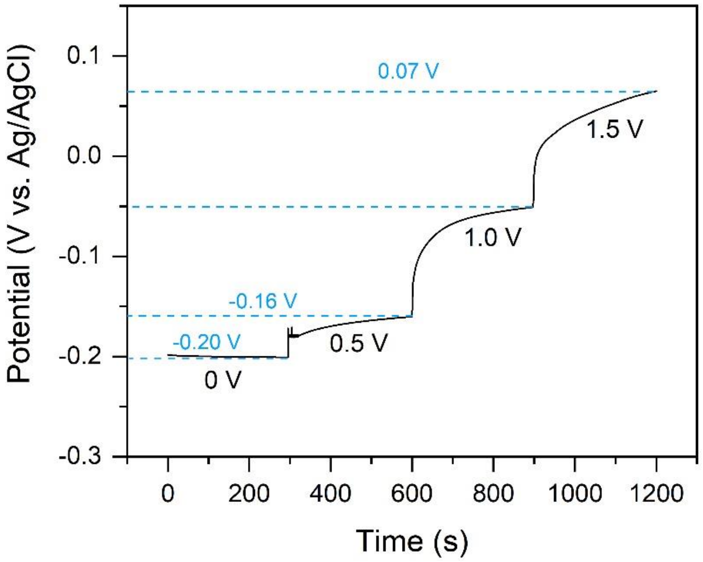

Supplementary Fig. 16. Surface potential of CNTs-PA membrane vs. the reference electrode (Ag/AgCl) when using it as the anode at different voltages in the presence of 1000 ppm Na_2SO_4.

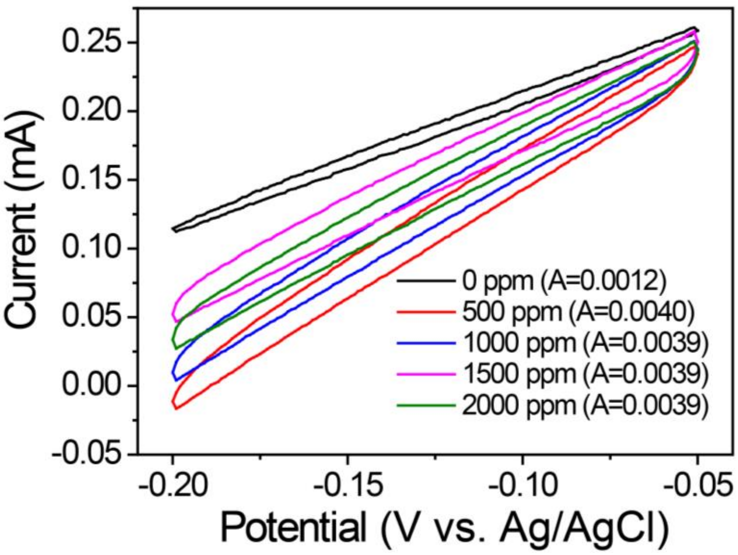

Supplementary Fig. 17. CV curves of the CNTs-PA membrane being used as an anode in the presence of Na_2SO_4 aqueous solution with different concentrations. The scanning potential range was determined by the open circuit potential measurement shown in Supplementary Fig. 16.

2.6 Pore size regulation of the CNT-PA membrane working as an anode

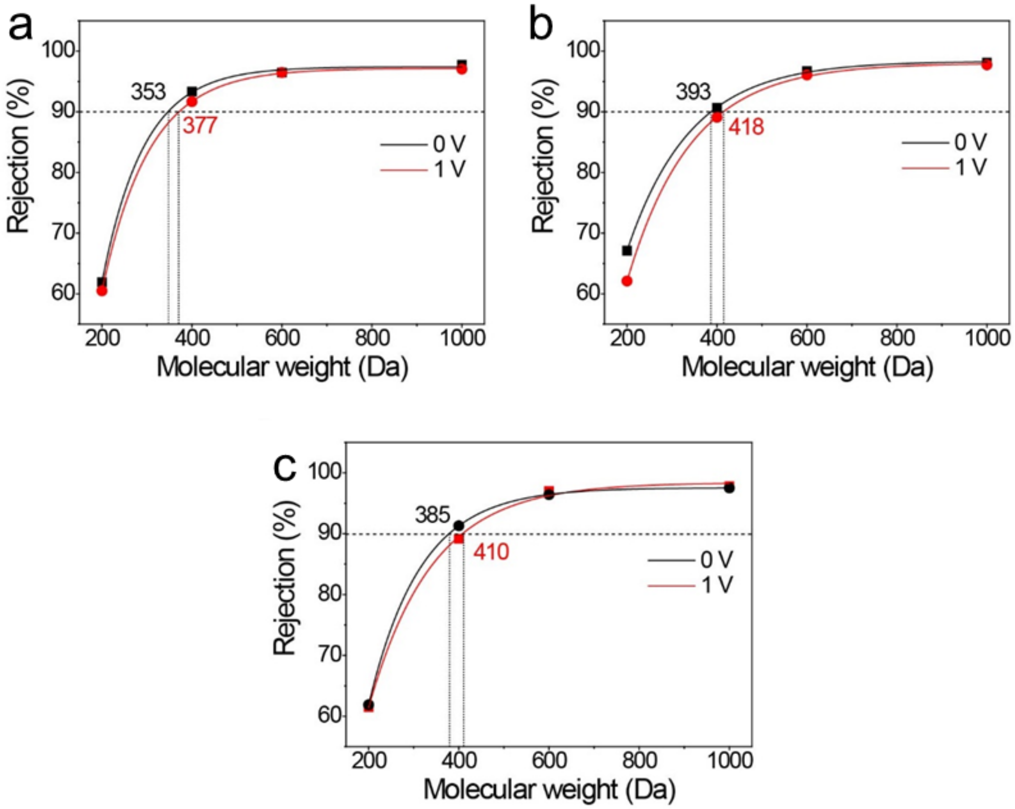

Supplementary Fig. 18. The rejection of PEG with different molecular weight by the CNTs-PA membrane being used as an anode at 0 V (black line) and 1V (red line). The measurement was performed in the presence of (a) 1000 ppm Na_2SO_4, (b) 1000 ppm $MgCl_2$, and (c) 1000 ppm LiCl. **Note:** Comparing the PEG rejection by the CNTs-PA membrane being used as an anode at the applied voltage of 0 V and 1 V, the MWCO shows a very slight difference. For instance, when the membrane worked in 1000 ppm Na_2SO_4 (Supplementary Fig. 18a), the MWCO at 0 V and 1 V was 353 Da and 377 Da, respectively. The MWCO difference is only 24 Da. In contrast, the MWCO difference is up to 114 Da when using the membrane as a cathode. The little change of MWCO is attributed to the negligible ion adsorption in the CNTs-PA membrane being used as an anode (Supplementary Fig. 17).

2.7 Pore size regulation of MPIP based CNTs-PA membrane by the applied voltage

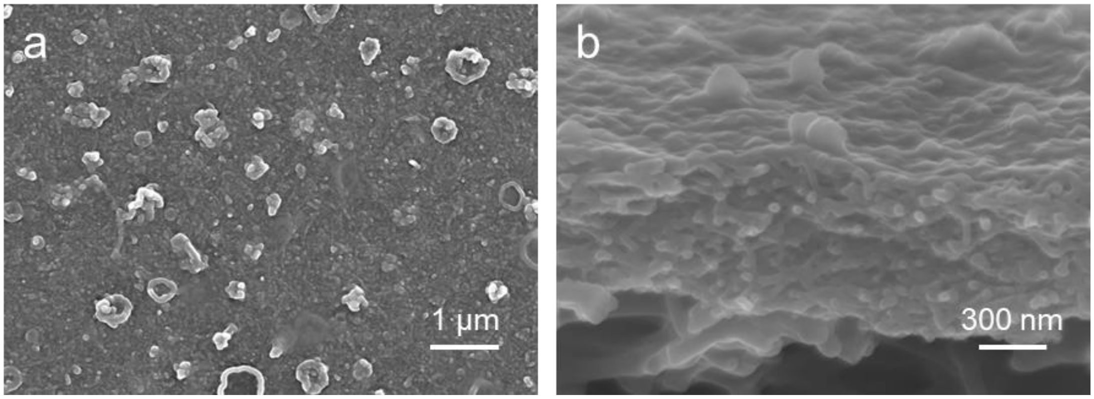

Supplementary Fig. 19. (a) Surface SEM image and (b) Cross-sectional SEM image of CNTs-PA membrane prepared using MPIP instead of PIP as amine monomer.

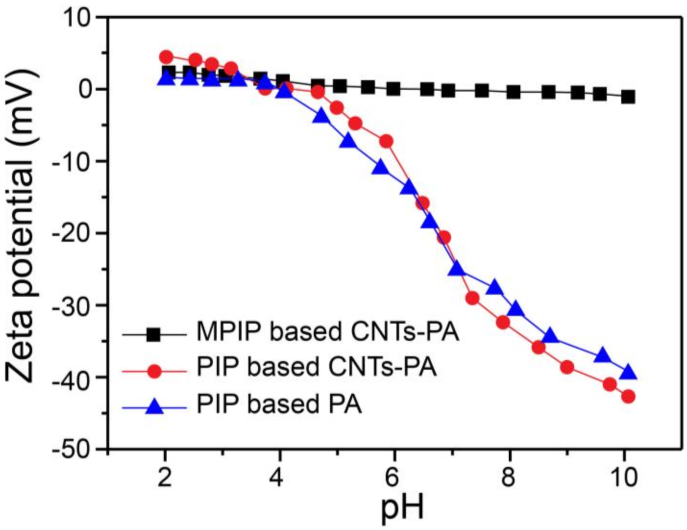

Supplementary Fig. 20. Surface zeta potential of PIP based PA membrane without CNTs (blue), PIP based CNTs-PA membrane (red), and MPIP based CNTs-PA membrane (black).

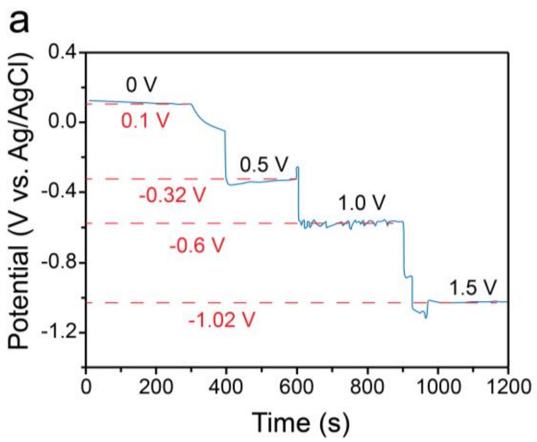

Supplementary Fig. 21. (a) Surface potential of MPIP based CNTs-PA membrane being used as cathode at different voltages in the presence of 1000 ppm Na_2SO_4. (b) Corresponding CV curves of the MPIP-based CNTs-PA membrane being used as the cathode in 1000 ppm Na_2SO_4.

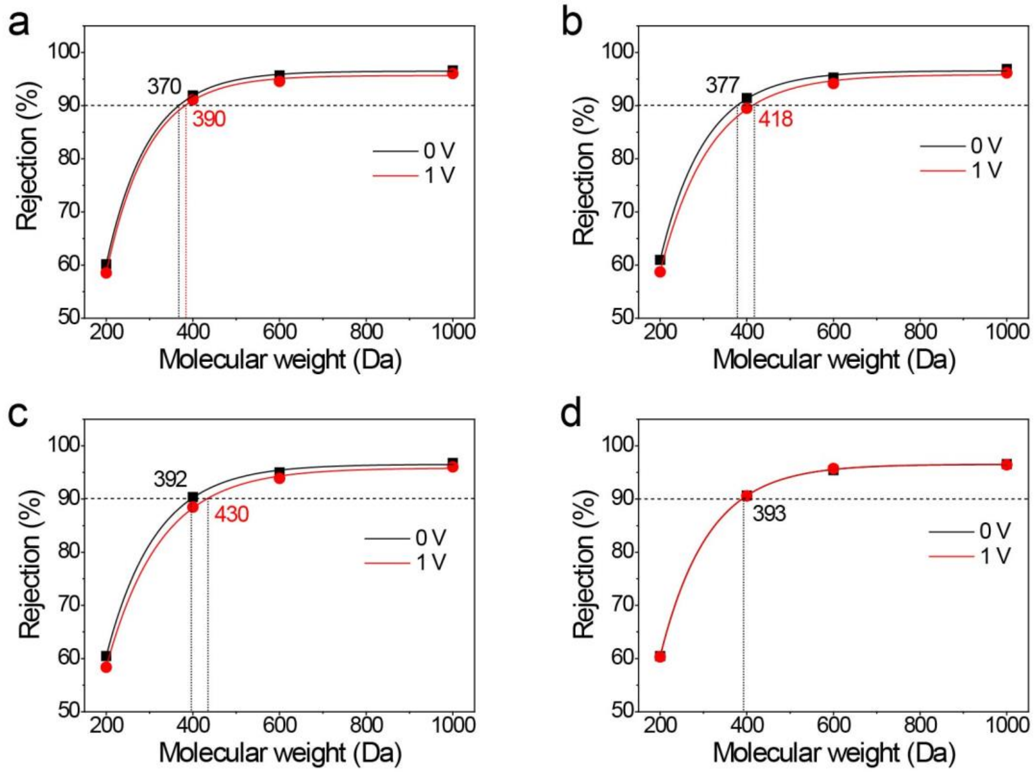

Supplementary Fig. 22. The rejection of PEG with different molecular weight by the MPIP-based CNTs-PA membrane being used as a cathode in (a) 1000 ppm Na_2SO_4, (b) 1000 ppm $MgCl_2$, (c) 1000 ppm LiCl electrolyte solutions, and (d) pure water.

Note: Compared with the PIP molecule, MPIP has lower activity but a stronger affinity towards hexane [4]. Therefore, more MPIP molecules will cross the interface and participate in the IP reaction with TMC, resulting in a thicker PA layer (Supplementary Fig. 19a). From the cross-sectional SEM image (Supplementary Fig. 19b), a similar topological structure with a PA layer embedded in the CNTs network was obtained. Meanwhile, due to the lower activity, more residual amine groups will exist in the PA layer, resulting in a slightly positive surface (Supplementary Fig. 20). In contrast, the PA layer from PIP shows a highly negative surface. Notably, the CNTs-PA membrane from PIP has similar surface charge to the PA membrane prepared using conventional ultrafiltration (UF) membrane as support without CNTs.

When the MPIP-based CNTs-PA membrane worked as a cathode in the presence of electrolyte solution (e.g., 1,000 ppm Na_2SO_4), the CV measurement detected a very lower ion adsorption than the one in the PIP-based CNTs-PA membrane (Supplementary Fig. 15). As a result, the MWCO of MPIP-based CNTs-PA membrane has a minimal shift when the membrane worked as a cathode at 0 V and 1 V either in an electrolyte solution or in pure water (Supplementary Fig. 22). This result suggests that the charge of PA layer plays an essential role in the pore size regulation by the applied voltage. Specifically, when the CNTs-PA membrane with a highly negative charge is used as a cathode in the presence of an electrolyte, more counter ions (e.g., Na^+) will be allowed to accumulate on the PA surface and enter the polymer chain network, resulting in a highly swelling polymer network and a large change of effective pore size. In contrast, if the CNTs-PA membrane has either a smaller number of negative charge or positive charge, the electrostatic exclusion effect will repel the counter ion like Na^+, resulting in little ion adsorption and weak regulation on the effective pore size by the voltage.

2.8 Pore size regulation of the CNTs-PA membrane by the voltage in pure water

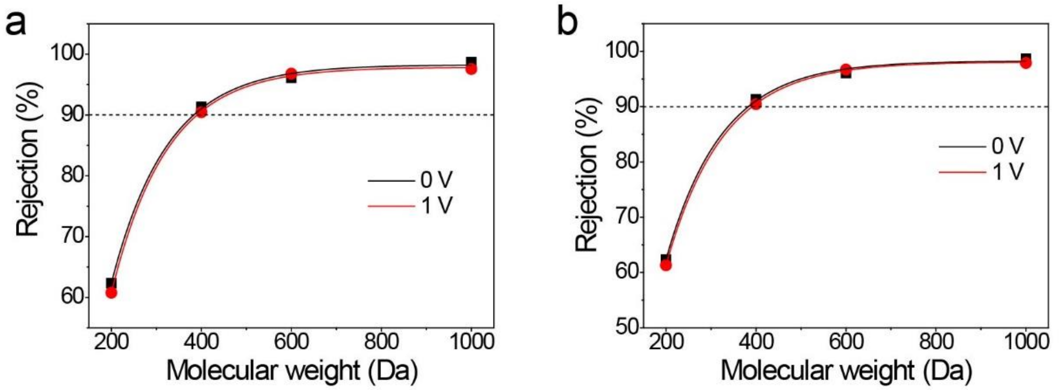

Supplementary Fig. 23. The rejection of PEG with different molecular weight by the CNTs-PA membrane being used as (a) a cathode and (b) an anode in pure water.

2.9 Partition coefficient of counter ions based on the Donnan equilibrium

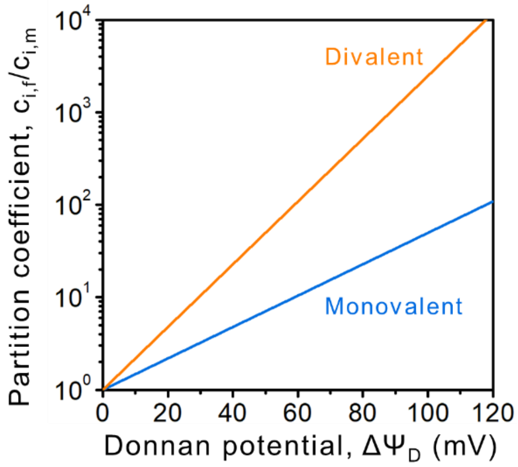

Supplementary Fig. 24. Partition coefficient of monovalent and divalent counter ions as a function of Donnan potential when only considering Donnan effect.

3 Supplementary Tables

Supplementary Table 1. Summary of the pore size of CNTs-PA membrane being used as cathode in the presence of different electrolyte solutions and applied voltages.

Electrode	Electrolyte solution (ppm)		Voltage (V)	Δc (mM)*	MWCO (Da)	r_p (nm)
**None**	Pure water	/	0	0	393	0.8004
**cathode**	Pure water	/	1	0	393	0.8115
	Na_2SO_4	500	0	102	361	0.7875
		500	1.0	631	434	0.8817
		1000	0	181	345	0.8095
		1000	0.5	439	426	0.8537
		1000	1.0	689	498	0.9130
		1500	1.0	893	523	0.9655
		2000	1.0	995	547	0. 9792
	$MgCl_2$	500	0	62	345	0.7873
		500	1.0	335	418	0.8793
		1000	0	13	393	0.7588
		1000	0.5	195	450	0.8485
		1000	1.0	429	491	0.8927
	LiCl	500	0	12	367	0.8176
		500	1.0	630	426	0.8995
		1000	0	18	385	0.7875
		1000	0.5	312	450	0.8667
		1000	1.0	710	466	0.9318
		1500	1.0	738	490	0.9665
		2000	1.0	871	531	0.9938

*Δc is the difference in ion concentration inside and outside the CNTs-PA membrane.

Supplementary Table 2. Desalination performance of CNTs-PA membrane being used as the cathode at different voltages for different electrolytes.

Electrolyte solution (ppm)		Voltage (V)	c_f (mM)	c'_f (mM)*	c_p (mM)	Rejection (%)	Flux (L m^{-2} h^{-1})
**Na_2SO_4**	500	0	3.52	5.65	0.06	98.43	48
	500	1.0	3.52	6.49	0.34	90.25	66
	1000	0	7.04	10.66	0.11	98.48	42
	1000	0.5	7.04	11.59	0.37	94.75	52
	1000	1.0	7.04	12.84	0.71	89.95	65
	1500	1.0	10.56	16.97	1.16	89.03	52
	2000	1.0	14.08	19.81	1.68	88.07	38
**$MgCl_2$**	500	0	5.26	6.90	2.74	47.98	50
	500	1.0	5.26	6.05	3.55	32.56	38
	1000	0	10.53	13.54	5.39	48.86	46
	1000	0.5	10.53	12.17	6.86	34.82	37
	1000	1.0	10.53	11.51	7.24	31.29	26
**LiCl**	500	0	11.76	14.35	8.74	25.67	62
	500	1.0	11.76	15.34	8.50	27.76	74
	1000	0	23.53	28.75	17.58	25.29	63
	1000	0.5	23.53	29.32	17.21	26.88	65
	1000	1.0	23.53	30.55	17.00	27.74	73
	1500	1.0	35.29	42.70	26.28	25.53	60
	2000	1.0	47.06	55.71	34.98	25.67	54

*c'_f is the interfacial feed concentration, and is estimated with the membrane theory assuming a mass transfer coefficient of 100 L m^{-2} h^{-1}, $c'_f = c_f\left[1 - R + R\exp\left(\frac{J_w}{k}\right)\right]$. C_f is the feed concentration of bulk solution and *Cp* is the concentration of permeate.

Note: A possible explanation for the flux decreases is the pore blocking induced by the adsorption of Mg^{2+} in the PA chain network. In detail, the high hydration energy of Mg^{2+} (437 kcal mol^{-1})[5,6] make them mainly present in the form of hydrated state in the PA chain network. The large steric hinderance of hydrated Mg^{2+}induces obvious pore block and causes in turn the flux decreases at 1 V. In comparison, the hydration energy of Na^+ is only 87.2 kcal mol^{-1}, leading to the easy

dehydration under pressure.[7] As a result, there is no obvious flux decreases when Na_2SO_4 and NaCl was used.

4. Supplementary References

1 Wang, Z. *et al.* Nanoparticle-templated nanofiltration membranes for ultrahigh performance desalination. *Nat. Commun.* **9**, 2014 (2018).

2 Lu, Y. *et al.* Two-dimensional fractal nanocrystals templating for substantial performance enhancement of polyamide nanofiltration membrane. *Pro. Natl. Acad. Sci. U. S. A.* **118**, e2019891118 (2021).

3 Teng, X. *et al.* High-performance polyamide nanofiltration membrane with arch-bridge structure on a highly hydrated cellulose nanofiber support. *Sci. China Mater.* **63**, 2570-2581(2020).

4 Gong, L. *et al.* Polyamide Nanofiltration Membrane from Surfactant-assembly Regulated Interfacial Polymerization of 2-Methylpiperazine for Divalent Cations Removal. *Chem. Res. Chin. Univ.* **38**, 782-789 (2022).

5 Nightingale Jr, E. Phenomenological theory of ion solvation. Effective radii of hydrated ions. *J. Phys. Chem.* **63**, 1381-1387 (1959).

6 Marcus, Y. Thermodynamics of solvation of ions. Part 5.—Gibbs free energy of hydration at 298.15 K. *J. Chem. Soc., Faraday Trans.* **87**, 2995-2999 (1991).

7 Shefer, I., Peer-Haim, O., Leifman, O. & Epsztein, R. Enthalpic and entropic selectivity of water and small ions in polyamide membranes. *Environ. Sci. Technol.* **55**, 14863-14875 (2021).
